# Case Report: IVIG causing bilateral papilledema and increased intracranial hypertension in patients with anti-TIF-1γ antibody-positive JDM

**DOI:** 10.3389/fped.2024.1433481

**Published:** 2024-11-13

**Authors:** Sarah Baluta, Ivana Stojkic, Kyla Driest, Christina Schutt

**Affiliations:** ^1^ Department of Pediatrics, Golisano Children’s Hospital, University of Rochester, Rochester, NY, United States; ^2^ Department of Pediatrics, Nationwide Children’s Hospital, Columbus, OH, United States

**Keywords:** juvenile dermatomyositis, anti-TIF-1γ antibody, intravenous immunoglobulin, increased intracranial pressure, myositis-specific antibodies

## Abstract

Juvenile dermatomyositis is a systemic autoimmune disease characterized by progressive proximal muscle weakness, pathognomonic rashes, and often the presence of myositis-specific antibodies. Consensus treatment plans for pediatric patients with juvenile dermatomyositis recommend steroids and methotrexate as initial therapy. Patients with anti-transcription intermediary factor 1 gamma (anti-TIF-1γ) antibodies tend to have more refractory disease requiring more aggressive treatment with intravenous immunoglobulin, which is typically well tolerated. We describe two pediatric patients diagnosed with anti-TIF-1γ antibody-positive juvenile dermatomyositis who developed persistent increased intracranial pressure following intravenous immunoglobulin treatment. These cases suggest a potential association between treatment with intravenous immunoglobulin and increased intracranial pressure, a side effect that is not readily known. The shared anti-TIF-1γ positivity in both patients may suggest a possible concern for intracranial hypertension among juvenile dermatomyositis patients with this myositis-specific antibody.

## Introduction

Juvenile dermatomyositis (JDM) is a rare, immune-mediated chronic inflammatory myopathy of childhood, typically present around age 7. It is characterized by progressive proximal muscle weakness, skin rashes, elevated muscle enzymes, and often the presence of myositis-specific antibodies. Both the adaptive and innate immune systems are thought to play crucial roles in the pathophysiology of JDM, with plasmacytoid dendritic cells, toll-like receptors, and the disruption of the interferon pathways being key players (1, 2).

Approximately 40%–60% of patients diagnosed with JDM have myositis-specific autoantibodies, which are associated with distinct clinical features that can help providers predict a patient's clinical phenotype and prognosis. The anti-transcription intermediary factor 1 gamma (anti-TIF-1γ) antibody is the most common antibody found in patients with JDM. Patients with anti-TIF-1γ antibodies tend to experience greater muscle weakness, skin involvement with Gottron papules, shawl sign, and poikiloderma and often receive more aggressive therapy (3).

In recent years, treatment approaches for children with JDM have evolved to mitigate the adverse effects of steroid monotherapy. The Childhood Arthritis and Rheumatology Research Alliance has published consensus treatment plans dependent on disease severity of disease that guide providers with updated treatment guidelines. Initial treatment for patients with moderate to severe disease includes a prolonged, gradual steroid taper and early initiation of methotrexate. For patients with active disease indicated by continued or worsened rash, persistently elevated muscle enzymes, and/or ongoing muscle weakness, the addition of intravenous immunoglobulin (IVIG) may be considered an additional therapeutic option (4–7).

IVIG is generally well tolerated, with most patients experiencing mild and transient side effects such as flushing, headaches, malaise, fever, chills, nausea, myalgias, rash, and fatigue. Rarely, more severe side effects such as aseptic meningitis, renal impairment, thrombosis, and hemolytic anemia have been reported. However, pretreatment with acetaminophen, diphenhydramine, and methylprednisolone prior to the infusion may help reduce side effects (8).

In this report, we present two cases of children diagnosed with anti-TIF-1γ antibody JDM who developed bilateral papilledema and increased intracranial pressure (ICP) upon escalation of their treatment regimen with IVIG for refractory disease. The development of papilledema and intracranial hypertension secondary to prolonged IVIG is sparsely documented in the literature, particularly in the context of pediatric JDM. Only a few case reports describe the development of bilateral optic disc edema and increased intracranial hypertension following IVIG administration (9, 10). We hope to raise awareness among providers of this potential side effect in patients requiring IVIG.

## Case 1

A 10-year-old girl was diagnosed with JDM based on progressive proximal weakness, polyarthritis, pathognomonic rashes, and laboratory evidence of myositis (Table 1). The diagnosis was further confirmed by the presence of anti-TIF-1γ and anti-P155/140 antibodies (Table 2), as well as an MRI of her bilateral thighs demonstrating symmetric myositis and mild subcutaneous (SQ) edema. Treatment was initiated with methotrexate [20 mg weekly SQ] and prednisone [60 mg (1.75 mg/kg) daily by mouth (PO)]. While her strength and arthritis improved rapidly, her skin disease persisted.

**Table 1:** Laboratory values for each case at the time of diagnosis, diagnosis of increased intracranial pressure, and 1 year post-IVIG discontinuation.

Laboratory test	Case 1				Case 2			
	Ref. range and unit	At the time of diagnosis	At the time of increased intracranial pressure	1 year post-IVIG discontinuation	Ref. range and unit	At the time of diagnosis	At the time of increased intracranial pressure	1 year post-IVIG discontinuation
WBC	4.5–12.5 × 10^3^/μl	5.6	6.1	4.7	3.5–11.0 × 10^3^/μl	15.5	8.6	8.1
HGB	12.0–16.0 g/dl	12.2	12.2	12.9	11.2–16.0 g/dl	12.7	12.0	10.1
HCT	36.0%–46.0%	37.5	35.4	37.9	34%–49%	38	38	34
PLT	142–508 × 10^3^/μl	358	331	296	150–450 × 10^3^/μl	347	350	426
Creatinine	0.5–0.8 mg/dl	0.28	0.44	0.42	0.20–0.70 mg/dl	0.27	0.44	0.47
BUN	5–18 mg/dl	10	12	9	6–10 mg/dl	15	9	10
Alk Phos	66–262 U/L	77	154	175	130–560 U/L	126	140	239
ESR	<13 mm/h	42	4	2	<20 mm/h	3	14	8
CRP					<8 mg/L	<3	5	<3
CK	<430 U/L	96	126	141	26–192 U/L	176	78	96
LDH	150–365 U/L	1,424	923	698	118–225 U/L	577	420	339
Aldolase	3.3–9.7 U/L	18.6	10.4	8.4	3.3–9.7 U/L	13.3	7.4	5.0
AST	15–50 U/L	21	47	66	0–35 U/L	57	41	25
ALT	<36 U/L	50	20	75	0–35 U/L	64	50	21

WBC, white blood count; HGB, hemoglobin; HCT, Hematocrit; PLT, platelet count; BUN, blood urea nitrogen, Alk Phos, alkaline phosphatase; ESR, erythrocyte sedimentation rate; CRP, C-reactive protein; CK, creatine kinase; LDH, lactate dehydrogenase; AST, aspartate aminotransferase; ALT, alanine aminotransferase.

**Table 2:** Myositis panel for both cases.

Antibody test	Ref. value	Case 1	Case 2
		Result	Result
SSA-52 IgG AB	0–40 AU/ml	0	0
SSA-60 IgG AB	0–40 AU/ml	7	0
Smith/RNP	0–40 AU/ml	9	
Ribonucleic protein (U1) IgG AB	0–40 AU/ml		3
Jo-1	0–40 AU/ml	1	1
PL-12 (alanyl-tRNA synthetase) AB	Negative	Negative	Negative
PL-7 (threonyl-tRNA synthetase) AB	Negative	Negative	Negative
EJ (glycyl-tRNA synthetase) AB	Negative	Negative	Negative
OJ (isoleucyl-tRNA synthetase) AB	Negative	Negative	Negative
Signal recognition particle (SRP) AB	Negative	Negative	Negative
KU AB	Negative	Negative	Negative
PM/Scl 100 IgG AB	Negative	Negative	Negative
Fibrillarin (U3 RNP) IgG AB	Negative	Negative	Negative
Mi-2 (nuclear helicase protein) AB	Negative	Negative	Negative
P155/140 AB	Negative	Positive	Positive
TIF-1γ (155 kDa) AB	Negative	High positive	Positive
SUMO activating enzyme (SAE1) AB	Negative	Negative	Negative
MDA5 (CADM-140)	Negative	Negative	Negative
Nuclear matric protein-2 (NXP2) AB	Negative	Negative	Negative

SSA, Sjogreb syndrome antibodies (A: SSa/Ro; B: SSB/La); AB, antibody; RNP, ribonucleoprotein antibody; OJ, (ant-OJ) anti-isoleucyl-transfer RNA synthetase; PM, polymyositis.

Monthly IVIG (1 g/kg) infusions were added to address her refractory dermatologic symptoms. Following her first IVIG infusion, she experienced a significant headache, necessitating frequent administration of analgesic medications. Despite the initial discomfort, her skin condition improved markedly. Therefore, IVIG was continued, with subsequent infusions administered at a slower rate over 12 h, which she tolerated well. She was successfully weaned off steroids 8 months after diagnosis.

After 8 months of IVIG therapy, the patient developed an intractable headache lasting for 9 days post-infusion and unresponsive to medications. MR brain imaging revealed indications of elevated ICP, including mild bulging of the globes at the insertion of the optic nerves, enlarged optic nerve sheaths, a partially empty sella, and decreased caliber of the left transverse venous sinus. An urgent evaluation by ophthalmology confirmed bilateral optic disc edema. A lumbar puncture demonstrated an elevated opening pressure of 36 cm H_2_O, with a closing pressure of 32 cm H_2_O. She was initiated on a Diamox taper for the management of elevated ICP, and her IVIG treatment was discontinued. Follow-up imaging 2 months later indicated resolution of the previously observed elevated ICP. Since discontinuing IVIG, she has experienced any recurrence of elevated ICP. She has subsequently maintained remission with methotrexate (20 mg weekly SQ) and hydroxychloroquine (200 mg daily PO).

## Case 2

A 7-year-old girl with progressive proximal muscle weakness and rash was diagnosed with JDM with positive TIF-1γ antibodies (Table 2). She was initially started on prednisone at 70 mg (2 mg/kg) daily PO and methotrexate at 25 mg weekly SQ. Despite aggressive therapy with high-dose prednisone and methotrexate, her skin remained refractory, with persistent elevations in muscle enzymes and inflammatory markers (Table 1). For her refractory disease, she was started on monthly IVIG [95 mg (2 g/kg)], premedicated with acetaminophen (15 mg/kg), diphenhydramine (1 mg/kg), and methylprednisolone (1,000 mg).

She showed significant improvement in her clinical symptoms, and her muscle enzyme levels returned to normal, although she developed intermittent nausea and headaches that would last 1–2 weeks after her IVIG infusions. Six months after initiating monthly IVIG infusions, an ophthalmology examination revealed elevated ICP and glaucoma. The elevated ICP was attributed to prolonged steroid use, and her steroids were aggressively tapered. A repeat ophthalmology examination showed progression to bilateral grade 2 papilledema with elevated ICP, despite her being on a 5 mg daily PO dose of prednisone, discontinuing methylprednisolone as a premedication for IVIG infusions, and discontinuing daily Bactrim.

MR brain imagining and MR brain angiography revealed significant signal loss along the left frontal sulci, indicative of superficial siderosis, along with mild irregularity, narrowing, and beading of the M2 segments of the bilateral middle cerebral arteries and P4 segments of the bilateral posterior cerebral arteries, raising concerns for intracranial vasculitis. In addition, mild protrusion of the optic disks into the posterior vitreous chambers was seen, confirming papilledema. She continued to experience increased intraocular pressure (IOP), which worsened to bilateral grade 3 papilledema on prednisone 2 mg daily PO (she was unable to tolerate discontinuation).

A lumbar puncture revealed an opening pressure of 45 cm H_2_O, confirming intracranial hypertension. Cerebrospinal fluid (CSF) analysis yielded no remarkable findings. Diamox was initiated for symptom management. Further investigations ruled out Lyme disease, thyroid dysfunction, and vitamin A and D toxicity.

Despite being on a low dose of prednisone and Diamox administered three times daily, her ophthalmology examination revealed progression from grade 3 to grade 4 papilledema. A second lumbar puncture, performed 6 weeks after the initial procedure due to worsening headaches and nausea, revealed an elevated opening pressure of 49 cm H_2_O, with no remarkable changes in the CSF.

Suspicion grew that her intracranial hypertension and papilledema might be attributable to the IVIG infusions. Consequently, a decision was made to gradually taper the patient off monthly IVIG over 2 months, after receiving a total of 11 doses, and transition her with mycophenolate mofetil. Approximately 2 months after discontinuing IVIG, an ophthalmology examination showed near-complete resolution of optic disc edema and elevated ICP. Follow-up imaging, performed 4 months after IVIG discontinuation, demonstrated no new areas of superficial siderosis, normalized cerebral arteries, and the absence of papilledema.

## Discussion

Intracranial hypertension is not a well-known adverse side effect of IVIG. Despite being used frequently for the treatment of autoimmune and inflammatory diseases, the exact mechanism of action of IVIG remains unclear. The most common adverse effects associated with IVIG are pyrexia, rigors/shivering, dyspnea, renal toxicity, hepatic issues, neurologic toxicity, gastrointestinal upset, and hematologic issues. Rarely, anaphylaxis, seizure, pulmonary edema, granulomatous uveitis, and dermatitis have also been reported. Headaches are the most frequent reaction to IVIG, occurring in 26%–61% of cases (11). Aseptic meningitis has also been well-documented as a side effect of IVIG, particularly in patients with a history of headaches and those receiving high doses of IVIG; the risk can be mitigated by using a slower infusion rate, lowering the initial IVIG concentration to 3% in the infuscate, ensuring prehydration, maintaining adequate fluid intake, and premedicating with acetaminophen and an antihistamine (12–14).

Interestingly, despite both patients receiving high-dose IVIG, neither had any predisposing risk factors, and both were premedicated with acetaminophen and an antihistamine. These patients were thought to potentially have aseptic meningitis, given their transient headache symptoms following infusions that were initially responsive to analgesic medications and a decreased infusion rate seen in case 1. It was not until after six to eight doses of IVIG that increased ICP in both patients was identified. This may indicate that this unknown side effect can occur in patients receiving prolonged high-dose IVIG treatment, which is not always the norm in patients without autoimmune conditions or those undergoing replacement therapy (12). However, reports of intracranial hypertension directly attributed to IVIG itself are scarce. In cases where intracranial hypertension coexists with IVIG administration, it has typically been associated with an underlying infection or neurologic condition, such as Guillain–Barre syndrome or multisystem inflammatory syndrome in children (MIS-C) (15–17).

In a case series involving four patients with intracranial hypertension and MIS-C who required ICU-level hospitalization and received IVIG treatment, only one patient developed intracranial hypertension after receiving IVIG, while the others had elevated ICP prior to receiving IVIG. The patient who developed increased ICP was identified 2 days after receiving IVIG with an ophthalmology examination significant for bilateral papilledema. The authors propose that this patient's increased ICP was secondary to MIS-C sequelae rather than from IVIG, as similar signs of increased ICP were observed in the other patients (16). Another case series describes a previously healthy 6-year-old child diagnosed with MIS-C who developed optic disc edema 1 week after receiving IVIG, per MIS-C guidelines. Subsequent examinations showed complete resolution of bilateral papilledema and right abducens nerve palsy after a steroid taper. The authors speculated that the patient’s increased ICP was due to the COVID-19 virus rather than a side effect of IVIG (17). These reports suggest an underlying neurological/infectious predisposition that may not have been fully evaluated in our patients. However, patients treated for MIS-C typically receive one dose of IVIG rather than repeated monthly doses given to our patients (18), which may explain why this side effect is not commonly observed or reported.

Central nervous system (CNS) complications are rarely reported in either juvenile or adult-onset inflammatory myositis. One case report identified CNS complications in 2 out of 193 pediatric JDM patients. Both patients had anti-NXP2 myositis-specific antibodies, yet neither developed increased ICP. Among JDM patients with CNS involvement, symptoms usually develop within 10 months of diagnosis, with seizures being the most common neurological symptom, followed by active cutaneous vasculitis (19). Therefore, it is less likely that the increased ICP observed in these two cases we described is from their disease process, and more likely represents a side effect of IVIG. However, this observation raises concern regarding an association with myositis-specific antibodies.

As IVIG continues to become readily used as a maintenance medication for autoimmune diseases, such as JDM, healthcare providers should continue to be aware of increased intracranial pressure as a potential side effect.

## Conclusion

This report of two JDM patients with anti-TIF-1γ antibodies who developed persistent intracranial hypertension following treatment with IVIG for JDM raises concerns about a potential association. While causality cannot be definitively established, these cases highlight the need for further investigation to determine whether intracranial hypertension is a rare side effect of IVIG or whether there is something underlying in the inflammatory milieu of these JDM patients that is predisposing them to this phenomenon. Clinicians should be vigilant for this complication in patients receiving IVIG, particularly those with autoimmune conditions, as it remains unclear if patients with JDM are at increased risk. Clinicians need to be aware of the potential risk of ICP in patients on IVIG and consider alternative treatment strategies in the setting of persistent intracranial hypertension, especially when no other etiology is identified.

## Data Availability

The original contributions presented in the study are included in the article/Supplementary Material, further inquiries can be directed to the corresponding author.
